# Detection of Sleep Biosignals Using an Intelligent Mattress Based on Piezoelectric Ceramic Sensors †

**DOI:** 10.3390/s19183843

**Published:** 2019-09-05

**Authors:** Min Peng, Zhizhong Ding, Lusheng Wang, Xusheng Cheng

**Affiliations:** 1 School of Computer Science and Information Engineering, Hefei University of Technology, Hefei 230601, China; 2 Anhui Province Key Laboratory of Industry Safety and Emergency Technology, Hefei 230601, China

**Keywords:** piezoelectric ceramic, sleep biosignals detection, respiratory rate, heart rate, wavelet analysis, ensemble empirical mode decomposition, dynamic smoothing

## Abstract

Physiological information such as respiratory rate and heart rate in the sleep state can be used to evaluate the health condition of the sleeper. Traditional sleep monitoring systems need body contact and are intrusive, which limits their applicability. Thus, a comfortable sleep biosignals detection system with both high accuracy and low cost is important for health care. In this paper, we design a sleep biosignals detection system based on low-cost piezoelectric ceramic sensors. 18 piezoelectric ceramic sensors are deployed under the mattress to capture the pressure data. The appropriate sensor that captures respiration and heartbeat sensitively is selected by the proposed channel-selection algorithm. Then, we propose a dynamic smoothing algorithm to extract respiratory rate and heart rate using the selected data. The dynamic smoothing can separate heartbeat signals from respiratory signals with low complexity by dynamically choosing the smooth window, and it is suitable for real-time implementation in low-cost embedded systems. For comparison, wavelet analysis and ensemble empirical mode decomposition (EEMD) are performed in a personal computer (PC). Experimental results show that data collected by piezoelectric ceramic sensors can be used for respiratory-rate and heart-rate detection with high accuracy. In addition, the dynamic smoothing can achieve high accuracy close to wavelet analysis and EEMD, while it has much lower complexity.

## 1. Introduction

Sleep information can be used to assess the health status of a person to a certain extent. Abnormal changes in sleep information are usually the forerunners of diseases. For instance, apnea and heart-rate variability appears in cardiovascular disease such as heart attack [1]. Thus, detecting the physiological information of people in the sleep state can help them manage their daily health and find the abnormal information [2,3], which helps to prevent tragedies from occurring.

Traditional sleep monitoring systems such as polysomnography (PSG) [4] are often used to detect the information of patients in hospitals or other institutions. They use sensors to collect data in various parts of the body such as the head, chest and abdomen of the patient. These intrusive measurement methods make the patient uncomfortable, which may probably affect the physiological indicators of the patient and get inaccurate information. In addition, when the patient moves or turns over, the sensors may fall off. Moreover, such sleep monitors are costly and complicated to operate, which limits their applicability in people’s lives.

In recent years, smart wearable devices such as wristbands and watches that can detect various physiological information of the human body have appeared [5,6,7]. However, such devices usually have low precision and need to direct contact with the skin. Since the bed is the basic requirement of sleep, using an intelligent bed or mattress to detect sleep biosignals has been the object of many research studies [8,9,10]. Unfortunately, most of these sensors used in intelligent beds or mattresses are costly or easy to damage. To meet the requirement of sleep biosignals detection for ordinary families, especially poor families, a low-cost and high-accuracy sleep biosignals detection system is required.

This paper proposes a low-cost intelligent mattress system to monitor sleep biosignals. Several piezoelectric ceramic sensors are placed under a normal latex mattress to sense respiration, heartbeat and body movements during sleep. The piezoelectric ceramic sensor has many advantages for physiological information detection. First, it can capture the pressure changes caused by small vibrations and convert them into electrical signals. Thus, through piezoelectric ceramic sensors, signals caused by breathing and heartbeat can be obtained. Second, it has much lower cost that other sensors such as piezoelectric film sensors and optical fiber sensors.

In this paper, data from a specific sensor is known as a data channel. Since sensors are deployed in different positions, parts of data channels cannot capture respiratory signals and heartbeat signals clearly because the corresponding sensors are far from the chest. On the other hand, to reduce the complexity, only one data channel is used for biosignals detection at a certain moment. Obviously, the selection of the data channel affects the detection accuracy. Experimental results show that the sensors close to the chest can capture respiratory signals and heartbeat signals more sensitively, and the corresponding data usually have larger variance. Thus, we propose a channel-selection algorithm by comparing the variance of data from different channels. Then, the physiological information is obtained through the data from the selected channel.

Wavelet analysis and EEMD are commonly used algorithms for time-frequency analysis. However, the complexity of wavelet transform and EEMD is high, and a powerful central processing unit (CPU) or additional chips are required to perform them in an embedded system. In this paper, we propose a dynamic smoothing algorithm to detect respiratory rate and heart rate with low complexity. By smoothing the original data with a dynamical smooth window, the waveform related to respiration can be obtained. Then, the heartbeat signals can be separated from other signals by another smoothing process. The experimental results show that piezoelectric ceramic sensors can capture respiratory signals and heartbeat signals sensitively, and the dynamic smoothing algorithm achieves a high accuracy close to wavelet analysis and EEMD.

The main contributions of this paper are summarized as follows.

- A sleep biosignals detection system based on piezoelectric ceramic sensors is built. By deploying 18 piezoelectric ceramic sensors arranged in two rows under the mattress, pressure data all over the mattress can be captured with low cost and high robustness.
- To select the most appropriate data channel which captures respiratory signals and heartbeat signals sensitively, we propose a channel-selection algorithm based on the comparison of the variance.
- For the purpose of implementing the sleep biosignals detection in a low-cost embedded system, the dynamic smoothing algorithm is proposed. In addition, time complexity and space complexity of the proposed algorithm are analyzed.

In the rest of this paper, we first review the related work in Section 2. Afterward, we introduce the framework of the sleep biosignals detection system in Section 3. In Section 4, sleep biosignals detection using wavelet analysis, EEMD, and dynamic smoothing is detailed. Then, experimental results are shown and analyzed in Section 5. Finally, we draw conclusions in Section 6.

## 2. Related Work

Electrical power can be generated from vibrations associated with movements of the human body through various sensors [11], hence respiration and heartbeat can be captured according to these slight vibrations. In recent years, there have been some works concentrating on detecting respiratory rate and heart rate using thin film piezoelectric sensors [12,13] and optical fiber sensors [14,15,16,17,18]. Based on the data measured by sensors, several methods are used to obtain respiratory rate and heart rate [7] using Ballistocardiography (BCG) [19], electrocardiogram (ECG) [20,21], photoplethysmogram (PPG) [22], and Seismocardiography (SCG) [19,23].

Thin film piezoelectric sensors can capture the slight vibration of the human body. Bu et al. [12] used a flexible piezoelectric thin film sensor to collect signals for respiration and heartbeat detection during sleep, and they employed empirical mode decomposition (EMD) which is proposed by Huang et al. [24] to extract signals corresponding to respiration and heartbeat from the measured data. Fujita et al. [13] developed a sensor made on a flexible thin film using polyvinylidene fluoride (PVDF) and poly dimethyl siloxane (PDMS) to monitor the heartbeat and respiration.

Optical fiber sensors can also sense the movements of the human body by measuring time of flight. Lau et al. [14] described a microbend fiber-optic sensor system to monitor respiratory signals in the magnetic resonance imaging (MRI) environment. Fajkus et al. [15] proposed a multichannel fiber-optic sensor system for basic vital sign monitoring. In [15], the Fourier series analysis was used to calculate respiratory rate and heart rate. Based on the optical coupling intensity ratio between an input and a set of aligned output optical fibers, Kam et al. [16] presented an aplastic optical fiber sensor for respiratory monitoring. Otis et al. used a new generation of mattress-based fiber-optic sensor for heart rate monitoring [17]. In [17], the peak searching algorithm, EMD, clustering and cepstrum methods [25] were compared for heart-rate detection. Aitkulov et al. [18] proposed a sensor based on the integration of a smartphone with a plastic optical fiber by using the flashlight as the source and the camera as a photodetector, and they applied the Fast Fourier Transformation (FFT) to detect heart rate.

Besides thin film piezoelectric sensors and optical fiber sensors, some other sensors have been used for sleep biosignals detection. Jia et al. [9] deployed a commercial off-the-shelf analog geophone under the mattress to monitor the user’s heartbeats during sleep, and sample auto-correlation function (ACF) was used to extract the periodicity of the captured signals and obtain the heart rate. Perez-Macias et al. [10] presented an automatic snore detection method using an electromechanical film transducer (Emfit) signal. Nayaka et al. [26] involved the use of a smart bed system to detect physiological information of the human body. The system inserts a pressure sensitive sensor between the wheel and the bed to obtain various physiological information of the human body. Lee et al. [22] designed a non-contact physiological detection pillow, in which the PPG sensor module and the capacitor coupled-ECG (CC-ECG) sensor module were embedded.

In contrast to the above research, we use piezoelectric ceramic sensors [27] which have higher stability and lower price to capture respiratory signals and heartbeat signals. In addition, although algorithms such as EMD have high accuracy, their complexity is too high to be performed in a low-cost embedded system. In this paper, we propose a dynamic smoothing method to detect respiratory rate and heart rate with low complexity, and the performance of piezoelectric ceramic sensors is confirmed by using the collected data to obtain respiratory rate and heart rate through wavelet analysis, EMD and dynamic smoothing.

## 3. The Framework of the Sleep Biosignals Detection System

**Figure 1:**
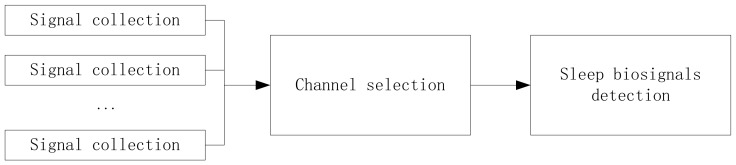
The framework of the sleep biosignals detection system.

To get the sleep biosignals with low cost, we build an intelligent mattress system based on piezoelectric ceramic sensors. In this system, respiratory rate, heart rate, and the number of turning over are considered, and the detection of sleep biosignals can be performed in a real-time manner. The whole system consists of signal collection, channel selection for appropriate signals, and sleep biosignals detection, as shown in Figure 1.

### 3.1. Signal Collection by Piezoelectric Ceramic Sensors

**Figure 2:**
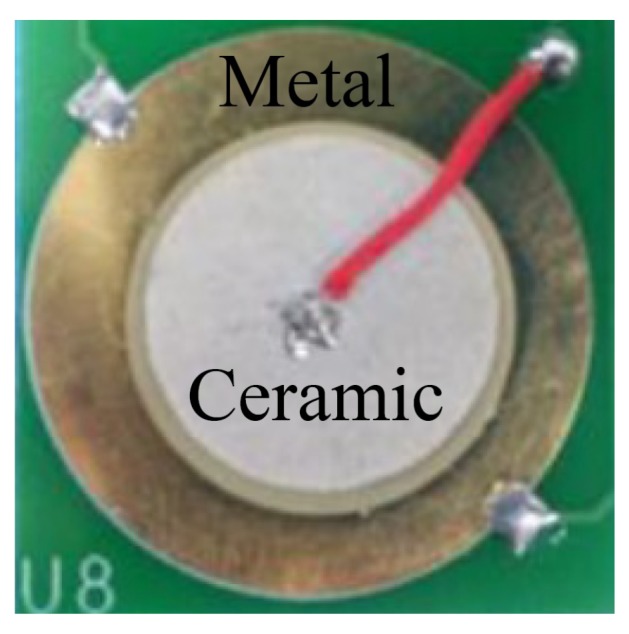
The piezoelectric ceramic sensor.

In this paper, purchased piezoelectric ceramic sensors are used for signal collection because of their low cost and high robustness. As shown in Figure 2, the piezoelectric ceramic sensor consists of a piezoelectric ceramic sheet and a metal sheet. The diameter of the piezoelectric ceramic sensor is 15 mm, and the thickness is 0.15 mm.

The internal dielectric of the piezoelectric ceramic sensor has positive piezoelectric effect and electrostrictive effect. And the deformation of the sensor will cause the change of the electric field.

(1) $$\begin{array}{c} T=cS-eE-gE^{2}\\ D=\epsilon E+eS+gES \end{array}$$

where *T* is mechanical stress, and *D* is electrical induction. *c*, *e*, and *g* are elastic, piezoelectric, and electrostrictive constants, respectively. *S* is the mechanical strain, *E* is the electric field intensity, and ϵ is the permittivity.

When the piezoelectric ceramic sensor suffers a slight deformation, electric charges will be generated on the surface of the sensor. By measuring the voltage, the changes in pressure on the sensor can be captured.

**Figure 3:**
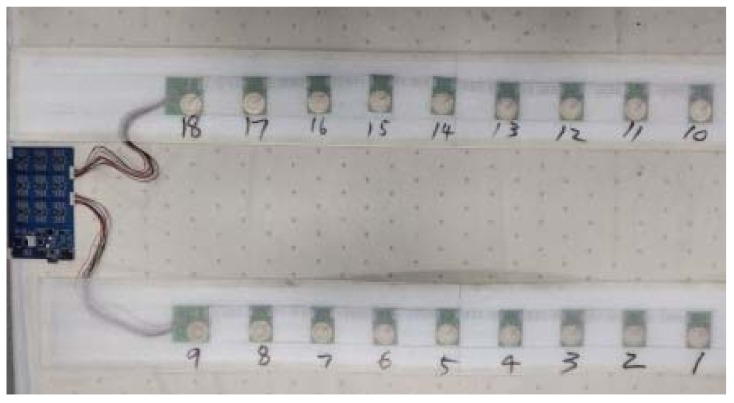
Piezoelectric ceramic sensors under the mattress and the embedded system.

Since the user may lie at different positions on the mattress, to collect data from almost the whole mattress, 18 piezoelectric ceramic sensors are deployed uniformly under the mattress, as shown in Figure 3.

When a person is sleeping on the mattress, the piezoelectric ceramic sensors under the mattress will suffer a slight deformation because of movements, respiration, and heartbeat. According to (1), the amplitude of the signals collected by the piezoelectric ceramic sensor is proportional to the vibration amplitude of the user’s body.

The original signals generated by piezoelectric ceramic sensors are usually slightly changed because the deformation caused by respiration and heartbeat is not obvious. To capture respiratory signals and heartbeat signals, amplifiers which can be implemented by discrete devices or in semiconductor chips are required. In this paper, we use 10x amplification to amplify the 0–300 mV signals generated by piezoelectric ceramic sensors to 0–3 V.

In addition, the analog voltage on the surface of the sensor should be converted into digital signals by analog-to-digital conversion (ADC). The sampling frequency of ADC in our system is 100 Hz.

### 3.2. Channel Selection for Appropriate Signals

**Figure 4:**
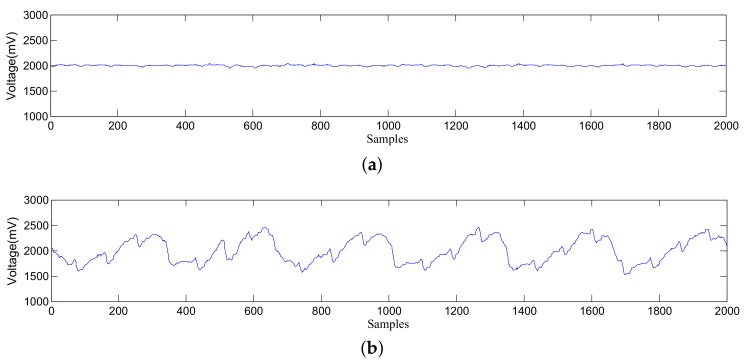
Illustration of data collected by different sensors. (**a**) Data from a sensor with weak signals; (**b**) Data from a sensor with strong signals.

Sensors are distributed in different positions, hence data collected by sensors are various. Figure 4 shows an illustration of data collected by different sensors. In the figure, there are 100 samples per second since the sampling frequency of ADC is 100 Hz.

The detection algorithms will use data series with a specific length to obtain biosignals. Thus, the continuously data collected by sensors should be divided into lots of data series at first. And the duration of each data series is defined as the time period. For example, the time period in Figure 4 is 20 s.

As shown in Figure 4, if the fluctuation of data is too small, it is difficult to extract respiratory rate and heart rate. To detect respiratory rate and heart rate more accurately, an appropriate channel (i.e., the sensor which can capture respiratory signals most sensitively) should be selected [28]. Moreover, since the user may turn over during sleep, the appropriate channel probably changes. Thus, the channel-selection process should be implemented periodically.

Obviously, the sensors near the chest are easier to capture changes caused by respiration and heartbeat, and they will obtain data with large variance.

In this paper, the variance of data from the *i*-th channel in the *t*-th time period is defined as

(2) $$S_{t,i}=\frac{\sum_{n=1}^{N}{\left(x_{t,i}\left(n\right)-\mu \right)}^{2}}{N}$$

where $X=\{x_{t,i}\left(n\right)\}$ is the data series obtained from the *i*-th channel in the *t*-th time period, *N* is the length of the data series, and μ is the mean value of *X*.

The variance of data is used for channel selection. The channel with the maximum variance is selected as the appropriate channel in this time period.

(3) $$I_{t}=\arg\max_{i}\left\{S_{t,i}\right\},\;i=1,2,\ldots ,18$$

where $I_{t}$ is the selected channel in the *t*-th time period.

### 3.3. Sleep Biosignals Detection

In addition to respiration and heartbeat, the piezoelectric signals are affected by many other factors such as circuit noise and human’s actions. The circuit noise is mainly the alternating current (AC) noise with the frequency of 50 Hz or 60 Hz. Therefore, the Twin-T notch filter [29] is used. An effective attenuation at the given frequency can be obtained through the appropriate Twin-T design. After preprocessing and denoising, the respiratory rate and heart rate can be extracted using the data from the appropriate channel.

In the built intelligent mattress, all processes are performed in the embedded system, and the micro controller unit (MCU) is STM32L151 with 32 KB Flash and 32 MHz CPU. Thus, the complexity of the detection algorithm should be considered. In this paper, a dynamic smoothing algorithm is proposed to extract respiratory rate and heart rate with low complexity. Meanwhile, wavelet analysis and EEMD are performed on a PC to obtain respiratory rate and heart rate and evaluate the validity of data collected by piezoelectric ceramic sensors.

## 4. Sleep Biosignals Detection Using Wavelet Analysis, EEMD, and Dynamic Smoothing

Once the user is on the mattress, the sleep biosignals detection system will select the appropriate channel to detect physiological information. To obtain the sleep time and save power, a preprocess of leaving bed detection is introduced.

### 4.1. Leaving Bed Detection

In this paper, 18 sensors are used to collect signals caused by the user’s respiration and heartbeat. If there is no body on the mattress, all sensors have almost no deformation, and all collected data maintain a stable value or just have slight changes. On contrary, when a person is on the mattress, parts of sensors will produce data with great changes due to human movements, breathing, and heartbeat. According to this phenomenon, the variance of data can distinguish whether there is someone on the mattress.

In the *t*-th time period, the total variance of all channels is

(4) $$CS_{t}=\sum_{i=1}^{18}S_{t,i}$$

where $S_{t,i}$ is the variance of the data from the *i*-th channel in the *t*-th time period.

If $CS_{t}$ is larger than the threshold, it indicates that there is someone on the mattress. Otherwise, there is nobody on the mattress, and the following processes are not required.

### 4.2. Turning Over Detection

The frequency of turning over is important for evaluating the quality of sleep. To detect turning over, sensors are divided into several regions. Sensors in the region close to the chest will obtain stronger signals than others. If the user turns over, the region with the strongest signals will change.

Assume the *k*-th region consists of 6 channels (i.e., channel $k_{1}$ to channel $k_{6}$), namely $R_{k}=\left\{k_{1},k_{2},\ldots ,k_{6}\right\}$. Then, the total variance of this region can be calculated as

(5) $$E_{k,t}=\sum_{i\in R_{k}}S_{t,i}$$

In the *t*-th time period, suppose the *k*-th region has the maximum total variance. In the next time period, if the region with the maximum total variance is changed, turning over is detected.

### 4.3. Respiratory-Rate and Heart-Rate Detection

In this section, the respiratory-rate and heart-rate detection is introduced in detail. Typical signals from an appropriate channel are shown in Figure 4b. It can be seen that respiratory signals and heartbeat signals are mixed with circuit noise and signals caused by human movements. In order to extract respiratory rate and heart rate from the mixed signals, three different methods, i.e., wavelet analysis, EEMD, and dynamic smoothing, are used.

#### 4.3.1. Wavelet Analysis

Wavelet transform is a signal analysis method developed based on Fourier transform. In the Fourier transform, original signals are transformed from the time domain to the frequency domain. The collected data from the sensors are non-stationary time series, and the Fourier transform cannot show the features both in time domain and frequency domain comprehensively. Instead, the wavelet transform can decompose non-stationary signals into different components, and it can highlight the local characteristics of signals in both time and frequency domains. Therefore, the wavelet transform is a commonly used method to detect respiratory rate and heart rate.

(6) $$Wf\left(a,\tau \right)=\langle f(x),\psi (a,\tau )\rangle =\frac{1}{\sqrt{a}}\int_{-\infty }^{\infty }f\left(x\right)*\psi \left(\frac{x-\tau }{a}\right)dt,\;a>0$$

where ψ(a,τ) is the wavelet function, *a* is the scaling factor, and τ is the translation factor. By adjusting the scaling factor *a* and the translation factor τ, wavelets with different frequency widths can be obtained.

In this paper, Daubechies wavelets (dbN) [30], Coiflets wavelets (coifN) [31] and Symlets wavelets (symN) [32] are used, because they are similar to the heartbeat signals.

Because the original wavelet transform is too complicated, fast discrete wavelet transform methods such as Mallat [33] are proposed. The Mallat algorithm adopts the hierarchical decomposition method. First, the one-dimensional original signal is decomposed into $a_{1}$ and $d_{1}$, where $a_{1}$ and $d_{1}$ are the low-frequency and high-frequency components, respectively. Then the $a_{1}$ part is further decomposed into $a_{2}$ and $d_{2}$, so that detailed information can be obtained. The frequency of the *i*-th layer signal after the wavelet decomposition in this manner is $\left[f_{L},f_{H}\right]$. And

(7) $$f_{L}=\frac{F_{s}}{2^{i+1}}$$

(8) $$f_{H}=\frac{F_{s}}{2^{i}}$$

where $F_{s}$ is the sampling frequency of the original signal.

Among the collected signals, the respiratory signals are the most obvious. Thus, the respiratory rate can be obtained through a good reconstruction of various wavelet transforms. In the sleep state, the respiratory rate is 0.2 Hz–0.5 Hz (i.e., 12–30 times per minute). The sampling frequency of the original signals is 100 Hz, and the signals are decomposed into 10 scales ($a_{1}$–$a_{10}$). According to (7) and (8), the frequency range of $a_{7}$ is 0.39 Hz–0.78 Hz, and that of $a_{8}$ is 0.19 Hz–0.39 Hz. Then, $a_{7}$ and $a_{8}$ which cover the frequency range of the respiratory waveforms are selected for wavelet decomposition and reconstruction.

**Figure 5:**
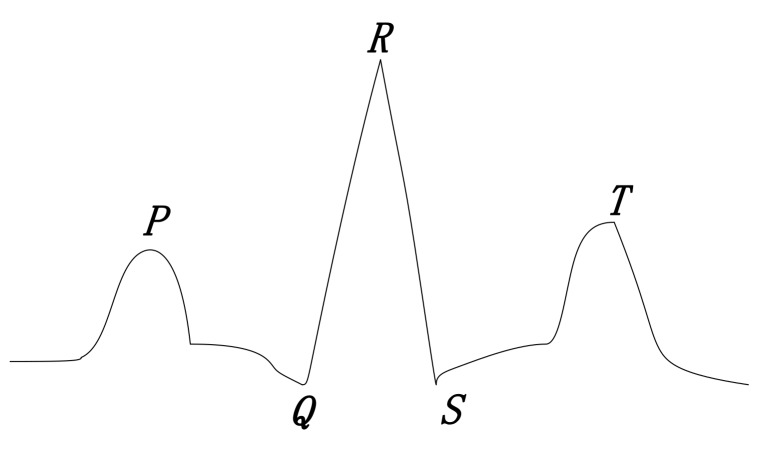
Illustration of standard ECG waves.

Similar to respiratory signals, heartbeat signals also fluctuate regularly but the intensity is much weaker than that of respiratory signals, and heartbeat signals are often hidden in the fluctuation of respiratory signals. As shown in Figure 5, a complete heartbeat cycle contains three waveforms, namely the P wave in the early stage, the maximum QRS wave in the middle-term fluctuation, and the T wave in the ending. The physiological structures of different human bodies are different, and the waveforms at different stages are slightly different.

The heartbeat frequency of the human body is 0.8 Hz–3 Hz (i.e., 48–180 times per minute). According to (7) and (8), the $a_{4}$–$a_{6}$ layer wavelets are selected for wavelet reconstruction to obtain heart rate. The time-frequency analysis of the signals by wavelet transform can represent the frequency change of signals in time, and the selection of the basis wavelet will affect the decomposition result. This paper uses multiple types of basis wavelets to process the original signals. Considering that the QRS wave has the largest fluctuation in the ECG waves, we select dbN, coifN and symN [30,31,32] which are approximate to the QRS wave as wavelets. Through the reconstruction experiments of various wavelets, db5, db6, sym6, and coif4 are selected. These wavelets are used for the final reconstruction, and heart rate will be extracted from the reconstructed signals.

#### 4.3.2. EEMD

The EMD algorithm [24] can decompose the non-stationary time series to several intrinsic mode functions (IMFs) and a residual value *R* with each IMF representing a frequency component.

The basis function of EMD is decomposed based on the real signals, while the basis function of Fourier transform is a sine-cosine function, and that of wavelet transform is the scaling and translation of the mother wavelet. Therefore, EMD decomposition depends on the data themselves and has self-adaptability, and it is an effective method for processing nonlinear and non-stationary time series.

The steps of EMD are as follows:

Step 1: The maximum and minimum values of the data series in the *i*-th time period $x_{i}\left(n\right)$ are obtained. Then, the upper envelope $U_{i}\left(n\right)$ and the lower envelope $L_{i}\left(n\right)$ are obtained by the cubic spline interpolation method [34] which is widely used in image processing. In addition, the average values of $U_{i}\left(n\right)$ and $L_{i}\left(n\right)$ can be calculated.

(9) $$m_{i}\left(n\right)=\frac{U_{i}\left(n\right)+L_{i}\left(n\right)}{2}$$

Step 2: Calculate the difference between the original series $x_{i}\left(n\right)$ and the average values $m_{i}\left(n\right)$.

(10) $$h_{i}\left(n\right)=x_{i}\left(n\right)-m_{i}\left(n\right)$$

Then, go to Step 3 if the condition of IMF is satisfied, otherwise, regard $h_{i}\left(n\right)$ as new data series and repeat Step 1 and Step 2.

Step 3: Calculate the residual value

(11) $$r_{i}\left(n\right)=x_{i}\left(n\right)-h_{i}\left(n\right)$$

Step 4: Regard $r_{i}\left(n\right)$ as new original data series and repeat the above operations.

At last, a total of *m* IMFs are obtained. The decomposition ends when the following criterion is satisfied:

(12) $$\sum_{n=1}^{N}\frac{{|h_{i,m-1}\left(n\right)-h_{i,m}\left(n\right)|}^{2}}{h_{i,m-1}{\left(n\right)}^{2}}\leq 0.2$$

where *m* is the number of IMFs and *N* is the length of data in this time period.

After EMD, $x_{i}\left(n\right)$ can be denoted as

(13) $$x_{i}\left(n\right)=\sum_{k=1}^{m}IMF\left(k\right)+R,$$

where *R* is the residual value.

EMD has good data decomposition characteristics, but it also has defects such as mode mixing. Mode mixing means that there are very different characteristic time scales in an IMF component, or adjacent characteristic time scales are included in different IMFs. To solve the problem of mode mixing in EMD, the ensemble EMD (EEMD) algorithm [35] was proposed. EEMD adds white Gaussian noise signals with different amplitudes to the original signals. The added white noise can compensate for the missing time scales, and then the signals have continuity at different time scales.

#### 4.3.3. Dynamic Smoothing

Since wavelet and EEMD have high complexity, we propose a simple dynamic smoothing method to obtain respiratory rate and heart rate. The basic idea of the dynamic smoothing is to smooth the data series with a dynamic window, and the length of the window depends on the recent respiratory rate or heart rate.

To get the signals corresponding to respiration, we smooth the data series with a smooth window of 2*W*+1, where $W=[F_{s}/\left(4r\right)]$. $F_{s}$ is the sampling frequency of the signals (i.e., 100 Hz), and *r* is the latest respiratory rate (in Hz). In the sleep state, respiratory rate is usually below 0.5 Hz (i.e., 30 times per minute). Thus, W≥50, and the initial value of *r* is set to 0.5 Hz.

Assume that the data series in the *i*-th time period are $X=\{x_{i}\left(n\right),n=1,2,\ldots ,N\}$, where *N* is the length of the data series. Then, the smoothed data series are

(14) $$x_{i}^{\prime }\left(n\right)=\left\{\begin{array}{ccc} \; & \frac{\sum_{m=n-W}^{n+W}x_{i}\left(m\right)}{2W+1} & W<n\leq N-W\\ \; & \frac{\sum_{m=1}^{2n-1}x_{i}\left(m\right)}{2n-1} & 1\leq n\leq W\\ \; & \frac{\sum_{m=2n-N}^{N}x_{i}\left(m\right)}{2N-2n+1} & N-W<n\leq N \end{array}\right.$$

According to $\left\{x_{i}^{\prime }\left(n\right)\right\}$, respiratory rate can be obtained by searching for the peaks and count the number of peaks.

Then, the data series with respiratory signals removed are approximated to the difference between the original data series and the smoothed data series.

(15) $$y_{i}\left(n\right)=x_{i}\left(n\right)-\alpha x_{i}^{\prime }\left(n\right)$$

where α is an amplification coefficient. The purpose of this coefficient is to eliminate the effect of the smoothing process. After smoothing, the average amplitude of $x_{i}^{\prime }\left(n\right)$ is less than that of $x_{i}\left(n\right)$. And α is estimated by comparing $x_{i}\left(n\right)$ and $x_{i}^{\prime }\left(n\right)$ around the peaks. Assume $x_{i}^{\prime }\left(n\right)$ is a peak value when n=k, then

(16) $$\alpha =\frac{\sum_{n=k-L}^{k+L}x_{i}\left(n\right)}{\sum_{n=k-L}^{k+L}x_{i}^{\prime }\left(n\right)}$$

where *L* is a value far less than *W*.

Similarly, the data series should be smoothed with a smooth window of 2w+1 to obtain heart rate, where $w=[F_{s}/\left(4h\right)]$ and *h* is the latest heart rate (in Hz). In the sleep state, heart rate is usually less than 2 Hz (120 times per minute). The initial value of *h* is set to 2 Hz.

The smoothed data series for heart-rate detection are

(17) $$y_{i}^{\prime }\left(n\right)=\left\{\begin{array}{ccc} \; & \frac{\sum_{m=n-w}^{n+w}y_{i}\left(m\right)}{2W+1} & w<n\leq N-w\\ \; & \frac{\sum_{m=1}^{2n-1}y_{i}\left(m\right)}{2n-1} & 1\leq n\leq w\\ \; & \frac{\sum_{m=2n-N}^{N}y_{i}\left(m\right)}{2N-2n+1} & N-w<n\leq N \end{array}\right.$$

At last, heart rate can be obtained by searching for the peaks in $\left\{y_{i}^{\prime }\left(n\right)\right\}$.

## 5. Experiments and Analysis

To confirm the validity of data collected by piezoelectric ceramic sensors and evaluate the sleep biosignals detection algorithms, experiments are performed in the laboratory under the same conditions as the bedroom. Piezoelectric ceramic sensors are arranged in two rows under the latex mattress, and the experimenters lie on the mattress.

Wavelet analysis, EEMD, and dynamic smoothing are used to obtain respiratory rate and heart rate using the data from the appropriate channel. While process the signals, three different lengths of time period are considered (i.e., 10 s, 30 s, and 60 s). As wavelet analysis and EEMD are too complex to be implemented in the STM32L151 embedded system, wavelet analysis and EEMD are run in a PC, while the dynamic smoothing algorithm is performed in the embedded system in a real-time manner.

When three algorithms are implemented to detect respiratory rate and heart rate, the heart rate is monitored synchronously using the contact medical ECG detector PC-80B, and the finger oximeter is used to synchronously measure the respiratory rate as a comparison. In the experiments, a total of 10 sets of results are obtained. By comparing the value obtained by detection algorithms and the true value measured by the finger oximeter or PC-80B, the accuracy can be obtained. In this paper, the accuracy is defined as

(18) $$Accuracy=\frac{\left|Value_{measured}-Value_{true}\right|}{Value_{true}}$$

where $Value_{true}$ is the value from the finger oximeter or PC-80B.

### 5.1. Respiratory Rate Tests

In the experiments with the time period of 60 s, the results of respiratory rate are shown in Table 1. The wavelet analysis has the highest accuracy and can exactly obtain respiratory rate in most cases. Meanwhile, EEMD and dynamic smoothing also achieve high accuracy. In the experiments with the time period of 30 s and 10 s, the results are shown in Table 2 and Table 3, respectively. With the time period of 30 s and 10 s, the accuracy decreases in all algorithms. In general, wavelet analysis and dynamic smoothing achieve better accuracy.

It is noted that the respiratory rate is updated every 10 s in all tests. The difference is that respiratory rate is calculated using the data in the nearest time period (i.e., 60 s, 30 s, and 10 s). Therefore, a shorter time period means that the changes in respiratory rate will be updated more quickly.

### 5.2. Heart-Rate Tests

Since heartbeat signals are very weak and superimposed on respiratory signals, heart-rate detection is much more difficult than respiratory rate detection. To evaluate the performance of heart-rate detection comprehensively, the signals obtained by different algorithms are shown at first, then the overall detection accuracy of each algorithm with different time periods will be shown.

**Figure 6:**
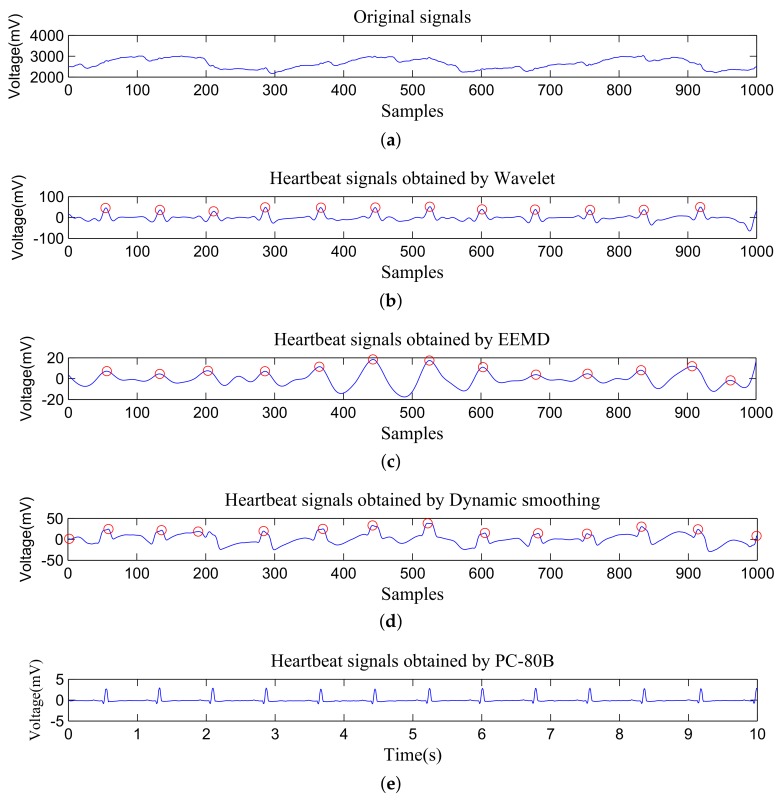
An illustration of original signals and heartbeat signals obtained by different methods. (**a**) Original signals captured by the piezoelectric ceramic sensor, (**b**) Heartbeat signals obtained by Wavelet analysis, (**c**) Heartbeat signals obtained by EEMD, (**d**) Heartbeat signals obtained by Dynamic smoothing, (**e**) Heartbeat signals obtained by PC-80B.

The heartbeat signals obtained by different algorithms and PC-80B are shown in Figure 6. Wavelet analysis can extract heartbeat signals very well, and the outline of ECG waves can be seen since dbN, symN, and coifN wavelets which are approximate to the QRS wave are used in wavelet analysis. The signals obtained by EEMD can find most of peaks correctly, but the ECG waveforms are missing since the signals are reconstructed by IMFs which mainly reflect the frequency. In the signals extracted by dynamic smoothing, most of peaks are found correctly too. Since the signals are obtained through smoothing, the ECG waveforms are also missing. Fortunately, in terms of heart-rate detection, all these algorithms are very accurate.

The accuracy of heart-rate detection with the time period of 60 s, 30 s, and 10 s is shown in Table 4, Table 5 and Table 6, respectively. In a word, the wavelet analysis has the highest accuracy, meanwhile, EEMD and dynamic smoothing also achieve high accuracy. The accuracy decreases with the decrease of the time period in all algorithms, and the accuracy of dynamic smoothing declines most slowly.

### 5.3. The Complexity Analysis

To evaluate the complexity of algorithms comprehensively, we take both time complexity and space complexity into account.

#### 5.3.1. Time Complexity

Time complexity of the Mallat wavelet transform was analyzed in [33]. It needs $N(3m+4+3/2log_{2}N)$ multiplications and $\left(N+3Nlog_{2}N\right)$ additions, where *m* is the wavelet length and *N* is the length or the data series. In this paper, 4 basis wavelets (i.e., db5, db6, sym6 and coif4) are used, and the time complexity is approximate to 4 times of the Mallat wavelet transform. Therefore, wavelet analysis in this paper needs $(52+6log_{2}N)N$ multiplications and $4(1+3log_{2}N)N$ additions.

In EEMD, the calculation of the upper and lower envelopes requires searching peaks and implementing interpolation. Comparing each point with its neighbors is required for such peak searching, and it needs 2N comparisons. Meanwhile, the interpolation for each point requires one addition and one multiplication, thus, *N* additions and *N* multiplications are required. Then, formula (9) needs a total of *N* additions and *N* multiplications. Therefore, Step 1 needs 4*N* comparisons, 3*N* additions and 3*N* multiplications. Step 2 and Step 3 need *N* additions, respectively. It is noted that these steps should be run several times. Assume that Step 1 and Step 2 should be sun *X* times for each IMF. In addition, the number of IMFs are *Y*. Finally, a total of (4N)XY comparisons, ((3N+N)X+N)Y additions, and (3N)XY multiplications are required. In the experiments, *X* is set to 100, and the average value of *Y* is 9.

In dynamic smoothing, the smooth process (14) for each point needs 2W additions and one multiplication, thus, a total of 2WN additions and *N* multiplications are required. *N* additions and *N* multiplications are required in (15), and (16) needs 4L additions and one multiplication. Similar to (14), (17) needs 2wN additions and *N* multiplications. Therefore, a total of 3N+1 multiplications and 2WN+2wN+N+4L additions are required. *W* and *w* are updated according to the latest respiratory rate and heart rate. And the average respiratory rate and heart rate are 0.3 Hz (i.e., 18 times per minute) and 1.25 Hz (i.e., 75 times per minute), respectively. Thus, the average values of *W* and *w* are 83 and 20, respectively. In addition, *L* is set to 10 in the experiments.

Furthermore, to calculate respiratory rate and heart rate, searching for the peaks is required in all algorithms. Thus, an additional 4N comparisons are required in each algorithm.

When the time period is 10 s, the length of the data series is 1000 (i.e., *N* = 1000). In this case, the quantitative comparisons of time complexity for three algorithms are show as Table 7. Since comparisons and multiplications require much more time than additions, the proposed dynamic smoothing algorithm has lower time complexity than wavelet analysis and EEMD.

#### 5.3.2. Space Complexity

Since STM32L151 has only 32 KB Flash, space complexity is more critical than time complexity. In the wavelet analysis, coefficient of $a_{1}$–$a_{10}$ for 4 wavelets should be stored, thus, 40 groups of data series are required. Moreover, the original data take a group of data series, and additional 2 groups of data series are required to store the reconstruction results. The original data series consist of *N* short integers, and each processed data series consist of *N* floats. Each integer and each float needs 2 bytes and 4 bytes of storage space, respectively. Therefore, it needs a total of 42N×4+2N bytes space.

In EEMD, $U_{i}\left(n\right)$, $L_{i}\left(n\right)$, $m_{i}\left(n\right)$, $h_{i}\left(n\right)$ and $r_{i}\left(n\right)$ are used in each round, and each of them consists of *N* data in float format. Considering that parts of data series can be covered after they have been used, at least 2 data series are required to be kept in the memory at the same time. For instance, $U_{i}\left(n\right)$, $L_{i}\left(n\right)$ must exist at the same time to obtain $m_{i}\left(n\right)$ according to (9). More importantly, each IMF should be stored. Therefore, it needs a total of (2+Y)N×4+2N bytes of storage space, where *Y* is the number of IMFs. In the experiments, the average value of *Y* is 9.

In dynamic smoothing, $x_{i}\left(n\right)$, $x_{i}^{\prime }\left(n\right)$ must be stored when calculating $x_{i}^{\prime }\left(n\right)$ because they will be used in (15), but in the computation of $y_{i}\left(n\right)$, they can be covered. Therefore, only 2 data series should be stored at the same time, i.e., a total of 2N×4 bytes of space are required. It is noted that the storage space of several variances such as *W* and *w* which just take several bytes are ignored in the analysis of space complexity.

When the time period is 10 s, the quantitative comparisons of space complexity for three algorithms are show as Table 8. Obviously, the memory requirements in wavelet analysis and EEMD are larger than the memory space of STM32L151.

In a word, wavelet analysis and EEMD cannot be implemented in the STM32L151 embedded system due to high space complexity, and the EEMD has far higher time complexity than dynamic smoothing.

All these experiments show that the data collected by piezoelectric ceramic sensors can be used for respiratory-rate and heart-rate detection. With an appropriate channel and a relative long time period, the results detected by wavelet analysis, EEMD, and dynamic smoothing are all with high accuracy. The wavelet analysis can obtain a very high accuracy in respiratory rate and heart rate; however, it has high complexity and cannot be implemented in the low-cost embedded system (e.g., STM32L151). On the other hand, the dynamic smoothing algorithm can also obtain a relative high accuracy which can meet the requirement of daily use. Since the dynamic smoothing method has much lower complexity than wavelet analysis and EEMD, it is suitable for detecting respiratory rate and heart rate in the embedded system in a real-time manner when the hardware cost is considered.

## 6. Conclusions

We built an intelligent mattress system based on piezoelectric ceramic sensors to detect sleep biosignals. To capture the signals caused by respiration and heartbeat more accurately, a channel-selection method was used. According to the physiological function characteristics of human body and the characteristics of piezoelectric ceramic sensors, wavelet analysis and EEMD can be used to obtain various life characteristics parameters such as respiratory rate and heart rate. To further decrease the complexity of the detection algorithm, a dynamic smoothing method was proposed. Experimental results show that piezoelectric ceramic sensors can capture signals caused by respiration and heartbeat. Moreover, respiratory rate and heart rate can be obtained accurately by wavelet analysis, EEMD, and dynamic smoothing. With the complexity considered, the proposed dynamic smoothing is more suitable for the low-cost embedded system.

**Ethics Statement:** All subjects gave their informed consent for inclusion before they participated in the study. The study was conducted in accordance with the Declaration of Helsinki.

## Figures and Tables

**Table 1:** The results of the respiratory rate test with the time period of 60 s.

Sets of Experiment	True Respiratory Rate	Wavelet		EEMD		Smoothing	
		Results	Accuracy	Results	Accuracy	Results	Accuracy
1	18	18	100%	17	94.44%	18	100%
2	18	18	100%	17	94.44%	18	100%
3	19	18	94.74%	18	94.74%	18	94.74%
4	19	18	94.74%	18	94.74%	18	94.74%
5	19	19	100%	18	94.74%	18	94.74%
6	19	19	100%	18	94.74%	18	94.74%
7	20	20	100%	20	100%	21	95.00%
8	20	20	100%	20	100%	21	95.00%
9	24	24	100%	22	91.67%	23	95.83%
10	24	24	100%	22	91.67%	23	95.83%
Average			98.95%		95.12%		96.06%

**Table 2:** The results of the respiratory rate test with the time period of 30 s.

Sets of Experiment	True Respiratory Rate	Wavelet		EEMD		Smoothing	
		Results	Accuracy	Results	Accuracy	Results	Accuracy
1	18	17	94.44%	17	94.44%	17	94.44%
2	18	20	88.89%	18	100%	19	94.44%
3	19	18	94.74%	18	94.74%	18	94.74%
4	19	19	100%	18	94.74%	19	100%
5	19	18	94.74%	17	89.47%	18	94.74%
6	19	20	94.74%	18	94.74%	19	100%
7	20	19	95.00%	20	100%	19	95.00%
8	20	20	100%	20	100%	21	95.00%
9	24	24	100%	14	58.33%	24	100%
10	24	24	100%	22	91.67%	22	91.67%
Average			96.25%		91.81%		96.00%

**Table 3:** The results of the respiratory rate test with the time period of 10 s.

Sets of Experiment	True Respiratory Rate	Wavelet		EEMD		Smoothing	
		Results	Accuracy	Results	Accuracy	Results	Accuracy
1	18	25	61.11%	8	44.44%	19	94.44%
2	18	18	100%	17	94.44%	19	94.44%
3	19	19	100%	20	94.74%	18	94.74%
4	19	17	89.47%	23	78.95%	19	100%
5	19	20	94.74%	18	94.74%	21	89.47%
6	19	18	94.74%	17	89.47%	19	100%
7	20	21	95.00%	20	100%	22	90.00%
8	20	19	95.00%	22	90.00%	23	85.00%
9	24	23	95.83%	21	87.50%	26	91.67%
10	24	24	100%	21	87.50%	25	95.83%
Average			92.59%		86.18%		93.56%

**Table 4:** The results of the heart-rate test with the time period of 60 s.

Sets of Experiment	True Heart Rate	Wavelet		EEMD		Smoothing	
		Results	Accuracy	Results	Accuracy	Results	Accuracy
1	70	70	100%	71	98.57%	68	97.14%
2	70	70	100%	71	98.57%	68	97.14%
3	75	70	93.33%	70	93.33%	68	90.67%
4	75	70	93.33%	70	93.33%	68	90.67%
5	70	71	98.57%	70	100%	69	98.57%
6	70	71	98.57%	70	100%	69	98.57%
7	73	73	100%	71	97.26%	71	97.26%
8	73	73	100%	71	97.26%	71	97.26%
9	73	72	98.63%	67	91.78%	67	91.78%
10	73	72	98.63%	67	91.78%	67	91.78%
Average			98.11%		96.19%		95.08%

**Table 5:** The results of the heart-rate test with the time period of 30 s.

Sets of Experiment	True Heart Rate	Wavelet		EEMD		Smoothing	
		Results	Accuracy	Results	Accuracy	Results	Accuracy
1	70	67	95.71%	67	95.71%	66	94.29%
2	70	72	97.14%	73	95.71%	79	87.14%
3	75	70	93.33%	69	92.00%	67	89.33%
4	75	70	93.33%	71	94.67%	69	92.00%
5	70	68	97.14%	67	95.71%	67	95.71%
6	70	73	95.71%	75	92.86%	72	97.14%
7	73	72	98.63%	67	91.78%	71	97.26%
8	73	76	95.89%	73	100%	72	98.63%
9	73	73	100%	70	95.89%	69	94.52%
10	73	78	93.15%	64	87.67%	65	89.04%
Average			96.01%		94.20%		93.51%

**Table 6:** The results of the heart-rate test with the time period of 10 s.

Sets of Experiment	True Heart Rate	Wavelet		EEMD		Smoothing	
		Results	Accuracy	Results	Accuracy	Results	Accuracy
1	70	80	85.71%	78	88.57%	75	92.86%
2	70	73	95.71%	74	94.29%	75	92.86%
3	75	68	90.67%	68	90.67%	77	97.33%
4	75	83	89.33%	83	89.33%	66	88.00%
5	70	71	98.57%	68	97.14%	68	97.14%
6	70	78	88.57%	79	87.14%	73	95.71%
7	73	73	100%	69	94.52%	69	94.52%
8	73	71	97.26%	68	93.15%	73	100%
9	73	73	100%	70	95.89%	74	98.63%
10	73	85	83.56%	62	84.93%	56	76.71%
Average			92.94%		91.56%		93.38%

**Table 7:** Quantitative comparisons of time complexity with *N*=1000.

Algorithms	Multiplications	Additions	Comparisons	Total Number
Wavelet analysis	111,795	123,589	4000	239,384
EEMD	2,700,000	3,609,000	3,604,000	9,913,000
Dynamic Smoothing	3001	207,040	4000	214,041

**Table 8:** Quantitative comparisons of space complexity with *N* = 1000.

Algorithms	Memory Requirements (Byte)
Wavelet analysis	170,000
EEMD	46,000
Dynamic Smoothing	8000
